# Types of Mastectomies and Immediate Reconstructions for Ipsilateral Breast Local Recurrences

**DOI:** 10.3389/fonc.2020.567298

**Published:** 2020-12-10

**Authors:** Pauline Simon, Julien Barrou, Monique Cohen, Sandrine Rua, Eric Lambaudie, Gilles Houvenaeghel

**Affiliations:** ^1^Department of Surgical Oncology, Institut Paoli-Calmettes, CRCM, Aix-Marseille University, Marseille, France; ^2^CNRS, INSERM, Aix Marseille Université, Marseille, France

**Keywords:** local breast cancer recurrence, mastectomy, breast reconstruction, implant, robotic surgery

## Abstract

**Purpose:** Ipsilateral-breast-local-recurrence (ILBLR) is a rare event with little data on immediate-breast-reconstruction (IBR). We report post-operative results of different types of mastectomy for ILBLR with or without IBR performed during a period of 40 months in order to analyze post-operative complications as main objective.

**Methods:** We analyzed mastectomies performed for ILBLR after initial breast conservative treatment from January 2016 to April 2019. The characteristics of patients, surgery, complication rate, postoperative hospitalization have been determined.

**Results:** Of the 207 mastectomies, 32.8% had an IBR: 31 nipple-sparing-mastectomy (NSM) and 37 skin-sparing-mastectomy (SSM) with 37 latissimus-dorsi-flap (LDF) IBR and 31 implant-IBR. Few reconstruction was performed for patients with body-mass-index ≥30 (OR = 0.214), infiltrating ductal carcinomas (OR = 0.272) and ASA-3 patients (OR = 0.254). In multivariate analysis, LDF-IBR was more often realized for NSM and for patients with BMI ≥25. The overall complication rate was 37.4%: 45.6 and 33.1% with and without IBR, respectively (*p* = 0.056). In multivariate analysis, BMI ≥25 (OR 2.02, *p* = 0.023), IBR (OR 1.9, *p* = 0.046) and tobacco (OR 2.17, *p* = 0.055) were correlated with higher risk of complications. There was no difference for Grade 2–3 complications rates for IBR and no IBR, respectively (14.7%: 10/68 and 9.3%: 13/139). In multivariate analysis, overall survival from date of mastectomy for local recurrence was significantly associated with interval time to local recurrence (OR 6.981).

**Conclusion:** Salvage mastectomy and IBR is a good choice for ILBLR, particularly using flap reconstruction. NSM can be considered as a good option in selected patients for ILBLR for NSM and/or LDFR.

## Introduction

The ipsilateral breast local recurrence (ILBLR) appears after breast conserving treatment (BCT), defined as breast-conserving surgery followed by whole-breast radiation therapy. Metastatic disease in the internal mammary, supraclavicular, infraclavicular, or ipsilateral axillary nodes was classified as a regional recurrence. In 2017, the rate of locoregional recurrences was 8.2% after BCT in very young woman (women <35 years old) with 11-year median follow-up (1). About 10% of patients of all ages will subsequently develop ILBLR (2). The annual incidence rate of isolated ILBLR in women diagnosed with an early invasive breast cancer (BC) was 0.6% (3). Incidence of isolated ILBLR (1.1–4.4% at 5 years) and interval between initial treatment and local recurrence vary significantly according to tumor subtypes (4).

ILBLR represents a challenge for clinicians because the management is not standardized. Very few studies have been dedicated to mastectomy with conservation of nipple area complex (NAC) for the treatment of ILBLR. The main objective of our study was to determine the effects of previous radiotherapy on surgical outcomes after mastectomy performed for ILBLR with or without immediate breast reconstruction (IBR). The secondary objective was to provide relevant data on complication rates and risk factors for complications according to type of mastectomy and type of IBR.

## Materials and Methods

### Study Design

This study was approved by institutional committee.

A retrospective, monocentric, cohort study was performed with the prospective data from patients who required a total mastectomy on a period of 40 months from January 2016 to April 2019. Among these patients, we analyzed mastectomies performed for ILBLR after initial BCT.

### Patients and Outcomes

The patients' anthropometric [body mass index (BMI)] and clinical data such as age, ASA status (American Society of Anesthesiology score), medical history (hypertension, diabetes), and tobacco use were recorded. Previous treatment for BC: sentinel lymph node biopsy (SLNB), axillary lymph node dissection (ALND), previous breast radiotherapy were also collected for risk factors analysis. Reconstruction methods and delay procedures were reviewed. ILBLR histology's were recorded with distinction in ductal and lobular invasive carcinoma, ductal carcinoma *in situ* (DCIS) and others (tubular, mucinous, medullary breast carcinoma). Post-operative acute complications were defined as complications that occurred until the third post-operative month. The rest were considered as chronic complications.

The acute complications were classified according to their nature: infectious (infection, fever), cutaneous (skin blistering, skin necrosis), hemorrhagic (hematoma, anemia) and complications related to lymph production (seroma, lymphedema).

The chronic ones were classified according to those related to the implant (fibrosis, ruptures, malposition), those linked to adjuvant treatment (inflammation, radiodermitis). Each type of complications was graded according to the Dindo classification (5).

For patients with breast implant we realized a nasal research of staphylococcus germ and pre-operative antimicrobial therapy for patients with nasal-germs. Then, per-operative antimicrobial-prophylaxis was systematically performed for all patients with IBR.

### Surgical Technique

Mastectomy and reconstructions were performed by a team of seven dedicated surgeons for breast and two plastic surgeons. The reconstruction method was determined by patient anatomy and preferences, and a variety of methods were used: latissimus dorsi-muscle flap (LDF) with traditional open technique or robotic technique (6) with or without implant, subpectoral implant, expander tissue, as judged appropriate.

The incision placements for NSM were preoperatively discussed and determined by breast and reconstructive surgeons together. NSM were conducted by breast surgeons using a standard procedure.

All patients were operated by total mastectomy with conservation of NAC when the tumor-to-NAC distance was at least 2 cm on preoperative radiological examinations. A retro-mammary biopsy was systematically performed, without extemporaneous analysis. Adjuvant radiotherapy may cause fibrosis and atrophy of the breast on the affected side and asymmetry of NAC position, without possibility of NAC conservation and without IBR indication in these cases.

### Periods

Two periods were established: P1 (years 2016–2017), P2 (years 2018–April 2019).

### Oncologic Outcome

Overall survival (OS) (death or last follow-up) and disease-free survival (DFS) (death or recurrence) from initial diagnosis and from mastectomy were analyzed with comparisons between ILBLR-free interval time (< or ≥ 60 months) and between patients with or without IBR.

### Statistics

For descriptive statistics, Mann-Whitney test was used to investigate continuous variables. The Fisher exact and the Pearson tests were used to investigate associations between two categorical variables. Multivariate analyses were performed using binary logistic regression in order to determined Odds Ratios (OR) with confidence interval (CI) 95% and *p*-values. OS and DFS were calculated with Kaplan Meier method and comparisons were evaluated using Log Rank test. Cox regression analysis was performed to determine significant factors associated with OS. A *p* ≤ 0.05 was considered statistically significant. All statistical analyses were performed using software SPSS 16.0.

## Results

### Patients

1,433 total mastectomies were performed, including 104 prophylactics, 1,122 for primary BC and 207 for ILBLR (13.4%) for 72, 1077, and 207 patients, respectively. IBR rates were 95.2% (99/104 mastectomies), 37.3% (419/1122), and 32.85% (68/207) for prophylactic, primary BC and ILBLR, respectively.

### ILBLR Mastectomy With or Without IBR

Characteristics of patients with total mastectomy for ILBLR with or without IBR were reported on Table 1. Patients without IBR were older than patients with IBR, with higher BMI, higher ASA status, higher mastectomy weight, higher breast cup size and with more ductal invasive tumors (Table 1). A strong correlation between BMI, cup size and mastectomy weight were observed (*p* < 0.01).

**Table 1:** Characteristics of patients diagnosed with ipsilateral breast local recurrence after conservative treatment of breast cancer (*n* = 207).

**Characteristics**	**Patients with IBR (*n* = 68)**	**Patients without IBR (*n* = 139)**	***p***
Age, *n* (%)			0.003
≤40	2 (2.9)	0 (0)	
41–50	12 (17.6)	12 (8.6)	
51–74	47 (69.1)	88 (63.3)	
≥75	7 (10.3)	39 (28.1)	
BMI, *n* (%)			0.002
<25	51 (75.0)	74 (53.2)	
25–29.9	14 (20.6)	35 (25.2)	
≥30	3 (4.4)	30 (21.6)	
Tobacco, *n* (%)			0.086
No	54 (79.4)	122 (87.8)	
Yes	14 (20.6)	17 (12.2)	
Diabetes, *n* (%)			0.238
No	66 (97.1)	130 (93.5)	
Yes	2 (2.9)	9 (6.5)	
ASA, *n* (%)			<0.0001
1	20 (29.4)	17 (12.2)	
2	44 (64.7)	90 (64.7)	
3	4 (5.9)	32 (23.0)	
Mastectomy, *n* (%)			<0.0001
NSM	31 (45.6)	1 (0.7)	
SSM	37 (54.4)	0 (0)	
Radical	0 (0)	138 (99.3)	
IBR type, *n* (%)			
Implant alone	31 (45.6)	–	
LDF	37 (54.4)	–	
Complication, *n* (%)			0.056
No	37 (54.4)	93 (66.9)	
Yes	31 (45.6)	46 (33.1)	
Reoperation, *n* (%)			0.151
No	60 (88.2)	130 (93.5)	
Yes	8 (11.8)	9 (6.5)	
Grade complication, *n* (%)			0.310
0	37 (54.4)	93 (66.9)	
1	21 (30.9)	33 (23.7)	
2	2 (2.9)	4 (2.9)	
3	8 (11.8)	9 (6.5)	
Grade Breast complication, *n* (%)			0.460
0	49 (72.1)	94 (67.6)	
1	10 (14.7)	32 (23.0)	
2	2 (2.9)	4 (2.9)	
3	7 (10.3)	9 (6.5)	
Years, *n* (%)			0.478
2016	20 (29.4)	39 (28.1)	
2017	25 (36.8)	42 (30.2)	
2018	19 (27.9)	41 (29.5)	
2019	4 (5.9)	17 (12.2)	
Bilateral mastectomy, *n* (%)			0.516
No	64 (94.1)	132 (95.0)	
Yes	4 (5.9)	7 (5.0)	
Mastectomy weight, *n* (%)			<0.0001
≤300	40 (58.8)	36 (25.9)	
>300	28 (41.2)	103 (74.1)	
Axillary, *n* (%)			
No	49 (72.1)	101 (72.7)	
SLNB	16 (23.5)	11 (7.9)	0.002
ALND	3 (4.4)	26 (18.7)	0.003
Missing	–	1 (0.7)	
Chemotherapy, *n* (%)			0.104
No	56 (82.4)	102 (73.4)	
Yes	12 (17.6)	37 (26.6)	
Endocrine therapy, *n* (%)			0.057
No	32 (47.1)	48 (34.5)	
Yes	36 (52.9)	91 (65.5)	
Cup size, *n* (%)			0.047
A–B	36 (52.9)	55 (39.6)	
≥C	32 (47.1)	84 (60.4)	
POHL, *n* (%)			<0.0001
≤3	35 (51.5)	126 (90.6)	
>3	33 (48.5)	13 (9.4)	
Histologies types, *n* (%)			<0.0001
DCIS	23 (33.8)	14 (10.1)	
NST	35 (51.5)	103 (74.1)	
Lobular	7 (10.3)	16 (11.5)	
Others*	3 (4.4)	6 (4.3)	
Interval chemotherapy, *n* (%)			0.601
≤60 days	10 (83.3)	29 (80.6)	
>60 days	2 (16.7)	7 (19.4)	

ALND, axillary lymph node dissection; BMI, body mass index; DCIS, ductal carcinoma in situ; IBR, immediate breast reconstruction; LDF, latissimus dorsi-muscle flap; Others*, tubular, mucinous, medullary breast carcinoma. POHL, post-operative hospitalization lengths; NST, nonspecific tumor; NSM, nipple sparing mastectomy, % percentage; SLNB, sentinel lymph node biopsy; SSM, skin sparing mastectomy.

In multivariate analysis few IBR were performed for patients with BMI ≥30 (OR = 0.214, *p* = 0.025, CI 95% 0.055–0.824), for ductal invasive carcinomas (OR = 0.272, *p* = 0.002, CI 95% 0.119–0.624) and for ASA-3 status (OR = 0.254, *p* = 0.057, CI 95% 0.062–1.044).

There were no significant differences of endocrine therapy rate and adjuvant chemotherapy rate and no significant difference of interval time between surgery and adjuvant chemotherapy between patients with or without IBR (median 42.5 vs. 43 days, mean 43.9 vs. 51.9 days, respectively) (Table 1).

### Patients With IBR for ILBLR Mastectomy

#### Types of Mastectomy

Types of mastectomy were 31 NSM (45.6%) and 37 SSM (54.4%). Any significant difference was observed between NSM and SSM for all analyzed criteria, except for histology of ILBLR with few rates of NSM for ductal and lobular invasive carcinomas in comparison with DCIS and others histology (Table 2). We doubled our NSM rate for ILBLR from 16% in 2016 to 29% in 2018.

**Table 2:** Characteristics of patients with immediate breast reconstruction after mastectomy for ipsilateral breast cancer local recurrence (*n* = 68).

**Characteristics**	**Total IBR (*n* = 68)**	**NSM (*n* = 31, 45.6%)**	**SSM (*n* = 37, 54.4%)**	***p***
IBR type, *n* (%)				0.063
Implant	20 (29.4)	9 (29.0)	11 (29.7)	
Expander	11 (16.2)	1 (3.2)	10 (27.0)	
LDF non autologous	4 (5.9)	3 (9.7)	1 (2.7)	
Autologous LDF	25 (36.8)	14 (45.2)	11 (29.7)	
LDF non autologous + implant	4 (5.9)	3 (9.7)	1 (2.7)	
Autologous LDF + implant	4 (5.9)	1 (3.2)	3 (8.1)	
Age, *n* (%)				0.153
≤40	2 (2.9)	2 (6.5)	0 (0)	
41–50	12 (17.6)	6 (19.4)	6 (16.2)	
51–74	47 (69.1)	22 (71.0)	25 (67.6)	
≥75	7 (10.3)	1 (3.2)	6 (16.2)	
BMI, *n* (%)				0.299
<25	51 (75.0)	26 (83.9)	25 (67.6)	
25–29.9	14 (20.6)	4 (12.9)	10 (27.0)	
≥30	3 (4.4)	1 (3.2)	2 (5.4)	
Tobacco, *n* (%)				0.101
No	54 (79.4)	22 (71.0)	32 (86.5)	
Yes	14 (20.6)	9 (29.0)	5 (13.5)	
Diabetes, *n* (%)				0.708
No	66 (97.1)	30 (96.8)	36 (97.3)	
Yes	2 (2.9)	1 (3.2)	1 (2.7)	
ASA, *n* (%)				0.131
1	20 (29.4)	11 (35.5)	9 (24.3)	
2	44 (64.7)	20 (64.5)	24 (64.9)	
3	4 (5.9)	0 (0)	4 (10.8)	
Cup size, *n* (%)				0.734
A-B	36 (52.9)	18 (58.1)	18 (48.6)	
C	25 (36.8)	10 (32.3)	15 (40.5)	
≥C	7 (10.3)	3 (9.7)	4 (10.8)	
Complication				0.252
No	37 (54.4)	15 (48.4)	22 (59.5)	
Yes	31 (45.6)	16 (51.6)	15 (40.5)	
Reoperation				0.259
No	60 (88.2)	26 (83.9)	34 (91.9)	
Yes	8 (11.8)	5 (16.1)	3 (8.1)	
Grade complication				0.717
0	37 (54.4)	15 (48.4)	22 (59.5)	
1	21 (30.9)	10 (32.3)	11 (29.7)	
2	2 (2.9)	1 (3.2)	1 (2.7)	
3	8 (11.8)	5 (16.1)	3 (8.1)	
Grade 2–3, *n* (%)				0.258
No	58 (85.3)	25 (80.6)	33 (89.2)	
Yes	10 (14.7)	6 (19.4)	4 (10.8)	
Mastectomy weight, *n* (%)				0.131
≤300 g	40 (58.8)	21 (67.7)	19 (51.4)	
>300 g	28 (41.2)	10 (32.3)	18 (48.6)	
Axillary, *n* (%)				
No	49 (72.1)	25 (80.6)	24 (64.9)	
SLNB	16 (23.5)	5 (16.1)	11 (29.7)	
ALND	3 (4.4)	1 (3.2)	2 (5.4)	
Years, *n* (%)				0.115
2016	20 (29.4)	5 (16.1)	15 (40.5)	
2017	25 (36.8)	14 (45.2)	11 (29.7)	
2018	19 (27.9)	9 (29.0)	10 (27.0)	
2019	4 (5.9)	3 (9.7)	1 (2.7)	
Chemotherapy, *n* (%)				0.270
No	56 (82.4)	27 (87.1)	29 (78.4)	
Yes	12 (17.6)	4 (12.9)	8 (21.6)	
Endocrine therapy, *n* (%)				0.176
No	32 (47.1)	17 (54.8)	15 (40.5)	
Yes	36 (52.9)	14 (45.2)	22 (61.1)	
Histologies types, *n* (%)				0.023
DCIS	23 (33.8)	16 (51.6)	7 (18.9)	
NST	35 (51.5)	11 (35.5)	24 (64.9)	
Lobular	7 (10.3)	2 (6.5)	5 (13.5)	
Others*	3 (4.4)	2 (6.5)	1 (2.7)	
POHL, *n* (%)				0.412
≤3	35 (51.5)	15 (48.4)	20 (54.1)	
>3	33 (48.5)	16 (51.6)	17 (45.9)	

ALND, axillary lymph node dissection; BMI, body mass index; DCIS, ductal carcinoma in situ; IBR, immediate breast reconstruction; LDF, latissimus dorsi-muscle flap; Others*, tubular, mucinous, medullary breast carcinoma. POHL, post-operative hospitalization lengths; NST, nonspecific tumor; NSM, nipple sparing mastectomy, % percentage; SLNB, sentinel lymph node biopsy; SSM skin sparing mastectomy; Autologous LDF, Latissimus dorsi-muscle with fat around muscle.

#### Type of Reconstruction

Reconstruction was realized with LDF in 37 patients (54.4%) and with implant in 31 patients (45.6%), 20 definitive breast implants and 11 expanders. For 8 patients, LDFR (latissimus dorsi-flap reconstruction) was associated with breast implant (8/37: 21.6%).

In univariate analysis, the first period and NSM vs. SSM were significantly associated with LDF reconstructions. More LDFRs were performed for patients with cup size ≥ C, but without significant difference. In multivariate analysis, LDFRs were more often realized during the first period, for NSM and for patients with BMI ≥25 (near significant result) (Table 3).

**Table 3:** Multivariate analysis to determined factors that influence the choice of LDF reconstruction.

**LDF vs. Implant**	***p***	**Odd ratio**	**CI 95%**	
			**Inferior**	**Superior**
BMI ≥25 vs. <25	0.053	3.717	0.98	14.0
P1 vs. P2	0.005	5.968	1.70	20.9
NSM vs. SSM	0.009	4.752	1.48	15.3

Other factors could determine the choice of LDF reconstructions: physic and professional activities, predominant side of activity, skin thickness and skin trophicity in relation with previous breast radiotherapy and patient's wishes.

#### Robotic Surgery

Forty three robotic procedures for 28 patients were performed among 68 mastectomies with IBR: 2 NSM with breast implant among 10 NSM with breast implant, 5 R-LDF reconstructions with SSM among 16 patients, 6 non-robotic NSM with R-LDF reconstruction and 15 R-NSM with R-LDF reconstruction among 21 NSM with LDF reconstruction.

#### Duration of Surgery

In univariate analysis, significant longer durations of surgery were reported for NSM vs. SSM, LDF-IBR vs. implant-IBR, LDF with implant vs. without implant, first period P1 vs. P2 and robotic surgery vs. no robotic surgery (Supplementary Data 1). In multivariate analysis, only LDF reconstructions in comparison with implant reconstructions were significantly associated with durations of surgery >180 min (OR 94, *p* < 0.0001, CI 95% 21–418).

#### Interval Time Before Chemotherapy

Median interval-time between surgery and chemotherapy when it was required was 43 days for patients with IBR (CI 95% 35–54) and 43 days for patients without IBR (CI 95% 41–62) (*p* = 0.417). IBR seems not to be a reason for delayed adjuvant treatment.

### Post-operative Outcome

#### Complications

##### All patients

Complication rate was 37.4% (77/206 patients) including all complications, 45.6 and 33.1% for IBR and no IBR, respectively (*p* = 0.056). Reoperation rate was 8.2% (17/207 mastectomies), 11.8 and 6.5% for IBR and no IBR, respectively (*p* = 0.151). There was no significant difference between IBR and no IBR for different grade of complications, with 14.7% (10/68) and 9.3% (13/139) Grade 2–3 complications for IBR and no IBR, respectively.

In univariate analysis significant factors known before surgery correlated with any complication were BMI (≥25), tobacco, IBR, mastectomy weight (>300 g), and surgery time (>180 min). In multivariate analysis, BMI ≥25 (OR 2.02, *p* = 0.023, CI 95% 1.1–3.7), IBR (OR 1.9, *p* = 0.046, CI 95% 1.0–3.6) and tobacco (OR 2.17, *p* = 0.055, CI 95% 0.98–4.80) were correlated with higher risk of complications.

##### For patients without IBR

Thirty three grade 1 complication (71.7% of all complications) were observed (28 seromas, 4 small skin necrosis and 1 hematoma), 4 grade 2 complication (2 skin blistering, 1 skin necrosis and 1 hematoma) and 9 grade 3 complication (5 hematoma, 2 infections and 2 skin necrosis) (Supplementary Table 1).

##### Patients with IBR

Complication rate was 45.6% including all complications (31/68) (Table 1). Twenty-one patients had grade 1 complication (67.7% of all complications), including 14 grade 1 dorsal complications corresponding to dorsal seromas which needed one or several punctures, 7 small breast skin blistering and 1 breast hematoma without reoperation. Two grade 2 breast complications were observed: 1 infection with medical treatment and 1 partial skin necrosis without reoperation. Eight reoperations (Grade 3) were required for two breast hematomas, two breast infections, three reoperations with implant loss, and one dorsal bleeding (Supplementary Table 1). The implant loss rate was 7.7% (3/39), 6.4% (2/31), and 12.5% (1/8) for breast implant or expander alone and implant associated with LDF, respectively.

In univariate analysis, more complications were observed with significant results for patients with tobacco use, cup size ≥C vs. A-B, LDF reconstructions vs. implant, duration of surgery >180 mn (Supplementary Data 2). In multivariate analysis, only LDF reconstructions in comparison with implant reconstructions were significantly associated with complications (OR 8.275, *p* = 0.009, CI 95% 1.7–40). Robotic surgery was not significant in comparison with non-robotic surgery.

Complication rate Grade 2–3 was 14.7% (10/68) but any factor was significantly associated with Grade 2–3 complications.

#### Post-operative Hospitalization Length

All patients: Post-operative hospitalization lengths (POHL) were higher for patients with IBR (medians 3.0 and 1.0) without any other significant factor.

##### Patients with IBR

In univariate analysis, significant higher POHL were observed for LDF reconstruction vs. implant (*p* < 0.0001), for BMI ≥25 (near significant *p* = 0.054) and for robotic surgery (*p* < 0.0001) without significant difference between NSM and SSM (*p* = 0.288). In multivariate analysis, only LDF reconstructions without implant and with implant were significantly associated with POHL >3 days (Table 4).

**Table 4:** Multivariate analysis of factors associated with surgery time, complications and POHL for patients with immediate breast reconstruction.

	***p***	**Odd ratio**	**CI 95%**	
			**Inferior**	**Superior**
**Surgery time ≤ vs**. **> 180 mn patients with IBR**				
Robotic surgery vs. no robotic	0.460	4.711	0.077	286.7
NSM vs. SSM	0.574	0.465	0.032	6.704
P2 vs. P1	0.163	0.166	0.013	2.074
LDF vs. implant	<0.0001	193.4	12.14	3080
**All complications patients with IBR**				
Tobacco yes vs. no	0.058	3.833	0.956	15.365
Cup size ≥C vs. A–B	0.087	2.689	0.867	8.337
LDF vs. implant	0.009	8.275	1.711	4.014
Robotic surgery vs. no robotic	0.263	0.413	0.088	1.946
**POHL** **≤3 vs**. **>3 days**				
BMI ≥ 25 vs. <25	0.599	1.446	0.365	5.725
Robotic surgery vs. no robotic	0.327	2.080	0.481	8.984
Implant/expander		1		
LDF	0.009	6.283	1.567	25.19
LDF and Implant	0.058	13.49	0.918	198.2

*BMI, body mass index; IBR, immediate breast reconstruction; LDF, latissimus dorsi-muscle flap; POHL, post-operative hospitalization lengths; NSM, nipple sparing mastectomy; SSM, skin sparing mastectomy; P1 and P2, period 1 and 2*.

##### Lipofillings

Lipofillings were realized in 12 patients at time of follow-up with 1 procedure for 10 patients and 2 and 3 procedures for 2 patients (median 188cc, mean 278cc, CI 95% 132–423cc): in 3 patients after breast implant reconstruction and 9 patients after autologous LDF reconstruction.

### Oncologic Outcome

Median follow-up were 198 months (CI 95% 175–207) from initial breast cancer diagnosis and 21 months (CI 95% 21–25) from the date of mastectomy for recurrence.

Median interval time between initial breast cancer diagnosis and local recurrence was 177 months (CI 95% 152–184): 47 patients (23.4%) with ILBLR-free interval <60 months and 154 patients (76.6%) with ILBLR-free interval ≥60 months. There was no significant association between ILBLR-free interval < or ≥60 months and IBR or no IBR (20% <60 months without IBR vs. 30.3%).

**Figure 1:**
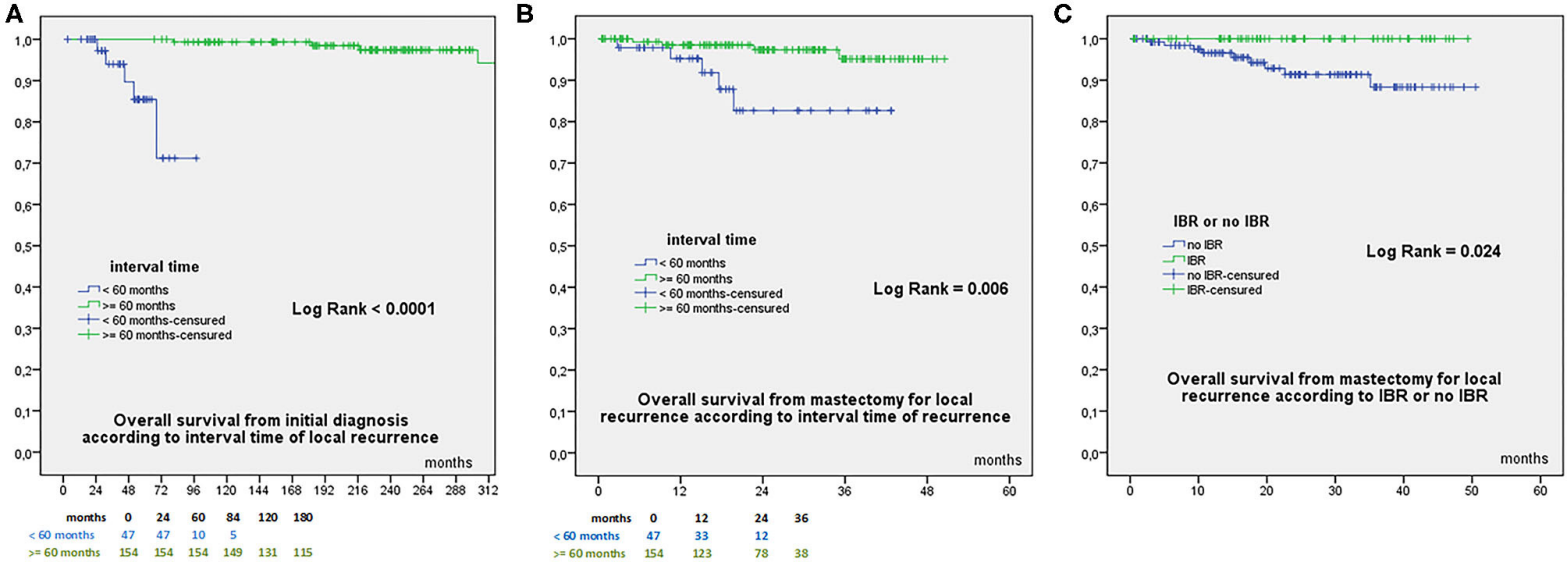
**(A)** Overall Survival from initial breast cancer diagnosis according to interval time to local recurrence < or ≥60 months. **(B)** Overall Survival from date of mastectomy for local recurrence according to interval time to local recurrence < or ≥60 months. **(C)** Overall Survival from date of mastectomy for local recurrence according to IBR or no IBR.

**Figure 2:**
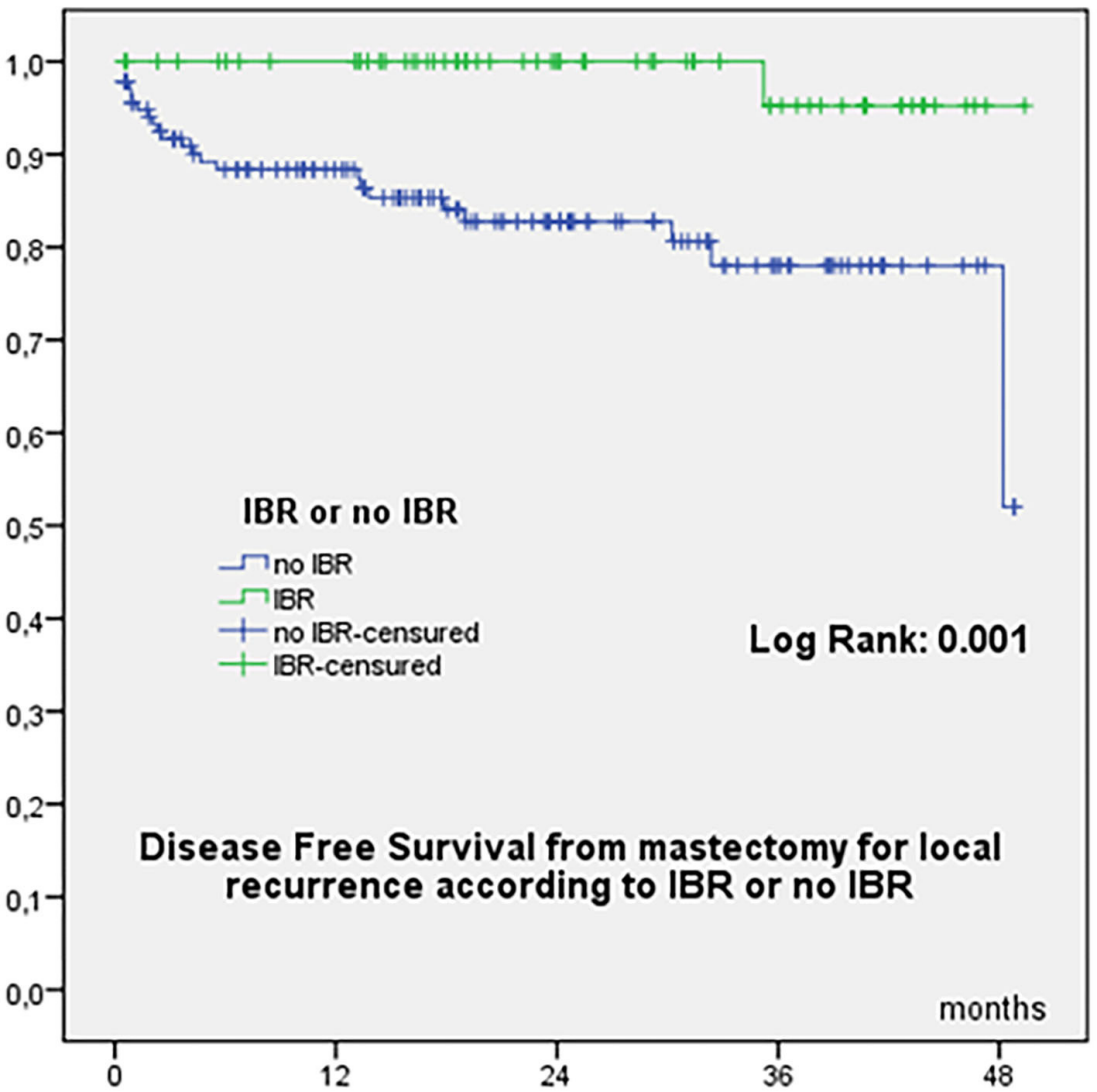
Disease-free survival from mastectomy for local recurrence according to IBR or no IBR.

OS from initial diagnosis was 97.8% at 60 months, 96.6% at 84 −180 months, and 94.7% at 240 months, with a total of 9 deaths (8 related to breast cancer evolution). OS from mastectomy for recurrence was 94.4% at 24 months and 92.5% at 38 months. OS according to time to local recurrence < or ≥ 60 months were significantly different from initial diagnosis (Figure 1A) and from mastectomy for recurrence (Figure 1B). OS from date of mastectomy for local recurrence according to IBR or no IBR were significantly different (Figure 1C). DFS from mastectomy for local recurrence according to IBR or no IBR were significantly different (Figure 2). In Cox regression analysis, OS from mastectomy for local recurrence was significantly associated with ILBLR-free interval (OR 6.981, CI 95% 1.83–26.6, *p* = 0.004) without significant association with IBR or no IBR (*p* = 0.959).

## Discussion

*ILBLR* remains a significant problem: The management of a BC recurrence is not standardized but a salvage mastectomy is recommended (7). The use of repeat BCT to treat ILBLR has been investigated with mixed results. Chen et al. evaluated 179 patients who underwent lumpectomy for treatment of ILBLR and only 21% of patients were documented to have re-irradiation (8). These patients had significantly worse 5 years OS (67%) than the patients who underwent mastectomy (78%). In a recent study, patients treated by lumpectomy alone had shorter OS and metastases-free survival than those with lumpectomy and re-irradiation (9). Lumpectomy alone seems therefore inappropriate for ILBLR (10). A new conservative treatment combined with brachytherapy or intraoperative radiotherapy (9, 11) or partial breast radiation (12) has been proposed to patients with tumors carrying good prognosis.

ILBLR is a rare event with an estimated rate of 0.5–1.5% per year after BCT for invasive carcinoma and an overall incidence ranging from 5 to 10% after 10 years (13, 14). The 5-year survival rate after mastectomy for ILBLR was around 60–86% (2, 15). However, survivals after ILBLR were significantly different with ILR-free interval <2 years or between 2 and 5 years in comparison with patients with ILR-free interval >5 years and for different tumor subtypes (4, 9, 16). A strong association between ILR-free interval and tumor subtypes had been reported (4). We reported here concordant results for ILR-free interval prognosis impact and no difference between patients with or without IBR in the multivariate model.

In the study by Frederick et al. (17), the IBR rate after mastectomy for ILBLR was 60%. Our rate of 32.8% reflects careful patient selection to achieve a high success rate and reasonably low complication rate. Moreover, the satisfaction with the cosmetic outcome strongly influenced quality of life and an unsatisfactory outcome after IBR was still considered a better condition than simple mastectomy (18).

### Type of IBR

For patients with previous radiotherapy for ILBLR, the latissimus dorsi-muscle nourishes and protects the thin skin. Robotic-LDFR was indicated in selected cases according to patient's choice and particularly for patients who don't want reconstruction with breast implant. In a large experience of Robotic-LDFR, the rate of Robotic-LDFR after previous radiotherapy was 44.4% (included mastectomy for ILBLR and mastectomy performed for primary BC after neo-adjuvant chemotherapy and radiotherapy) (19). In our study the majority (54.4%) of IBR after previous radiotherapy was performed by Robotic-LDFR.

### Type of Mastectomy With IBR

NSM is preferred over SSM in patients when the tumor is at a distance to the NAC. NSM can be proposed in selected cases of ILBLR (20). We reported a high rate of NSM for ILBLR during this recent period, 23.8% of NSM for ILBLR (20/84).

NSM has become increasingly common for therapeutic indications because of their cosmetic advantage (18) associated with better woman's body image and quality of life (21, 22), improved patient satisfaction and psychosexual benefits (23).

However, the potential disadvantage includes an increased risk of necrosis of the skin flaps and/or the NAC. Complications rates are difficult to compare with other studies in reason with the lack of consensus as to how to define the outcomes. In 2011, a systematic review revealed that the complications of necrosis, hematoma, seroma, and infection were defined in fewer than 20% of the 134 studies reviewed (24). This lack of consistency with respect to outcomes definitions makes it difficult to determine accurate complication rates for procedure.

### NSM and Outcomes: For Recurrent, Primary and Prophylactic Surgery

A little is known about the suitability and outcomes of NSM with IBR in the treatment of recurrent BC. The largest prospective one includes 982 NSM with IBR, and 49 for ILBLR (25). Overall complications were significantly more frequent in NSM with prior radiotherapy (21.7%) compared to those with no radiotherapy (10.2%). Prior radiotherapy increases the overall rates of skin necrosis (11.5 vs. 4.5%) and nipple loss (11.6 vs. 0.9%). Complications that required surgical intervention were seen in 18.8% in the cohort with prior radiotherapy vs. 7.1% in the cohort with no prior radiotherapy. In a recent study of Lee et al. (26), prior BCT did not contribute to higher surgical complications rate in patients who received NSM and IBR after ILBLR except for the total nipple loss with a higher rate in the group with prior radiotherapy compared with the group with NSM for primary BC (0.8 vs. 11.1%, *P* = 0.041).

In our study, there was no significant difference between NSM and SSM for different grade of complications or for the rate of complications, but we identified two independent risk factors associated with higher risk of complications: tobacco and BMI. These factors are all available before surgery, which may allow surgeons modify the reconstruction plans to reduce the complication rate. Thermal-injury (27) is also a determining factor of complication: our group use plasma blade instead of traditional electro cauterization to reduce thermal injury during NSM (28). This report shows that NSM for ILBLR may be considered as a treatment option with an acceptably low rate of complications and satisfactory short-term outcomes for appropriately selected patients.

Recently, NSM has become a favorable option for primary BC surgery when performed in selected patients (29). In a recent systematic review of the oncological safety of primary NSM, the overall complication rate was 22.3% and the nipple necrosis rate was 5.9% (30). Importantly, they found that the rates of complications, including nipple necrosis, decreased over time which was attributed to improving surgeon expertise. NAC necrosis is a particular issue when patients have risk factors such as smoking, young age, high BMI, peri-areolar skin incision for mastectomy (31–35). The identification of such risk factors could help clarify patient selection criteria.

About complications of prophylactic NSM (P-NSM), the largest series of 3716 P-NSM was published by Muller et al. (36). They reported an average overall complication rate at 20.5, 8.1% NAC necrosis and 7.1% necrosis of the cutaneous skin flaps.

A recent retrospective review (37), one of the largest studies on genetic carriers undergoing NSM, examined outcomes of 397 NSMs BRCA1/2 carriers. Flap necrosis occurred in 10 (2.5%) and NAC loss occurred in 7 (1.8%) breasts, three dues to cancer involvement (5.8%) and four from necrosis. In our study the rate of cutaneous necrosis after IBR for ILBLR was 1.4% (1/68), quite similar with these studies on P-NSM.

Overall complication rates were low and comparable to non-carrier populations (32).

The use of robotic minimally invasive surgery has also shown a low complication rate. In the study of Sarfaty (38), a total of 33 women underwent 63 robotic P-NSM with IBR by pre-pectoral implant. In a short term (21 days), there were no cases of major/minor mastectomy skin flap or NAC necrosis, three postoperative infections and one prosthesis explant.

In our study, there were no significant difference between the rate of overall complications (*p* = 0.056), complication grade (0.310) and reoperation rate (*p* = 0.151) with or without IBR. Previous studies have shown that the complication rate are still higher in radiated breasts, even with autologous tissue (39, 40). Our results can be explained by a rigorous selection of patients with IBR. This may be also attributed to differences in technique, newer implants or differences in radiotherapy protocols. Our results are in agreement with a recent publication by Lee et al. showing that prior BC did not contribute to higher surgical complications in patients who received NSM and IBR as a salvage procedure after ILBLR (26). However, it is difficult to conclude on the implication of IBR on the complications of a mastectomy for ILBLR because there is no study comparing the complications of a mastectomy for recurrence with and without reconstruction.

About the type of breast reconstruction, only LDF reconstructions in comparison with implant reconstructions were significantly associated with complications. LDF is first line for patients who have high-risk comorbidities such as diabetes, obesity, or tobacco use and for patients whose breasts have been radiated (41).

### Complications, Time of Surgery, Anesthesia and Robotic Surgery

We reported our experience of 43 robotic procedures for 28 patients among 68 mastectomies with IBR. It was a robotic NSM with or without LDF for ILBLR with a standardized surgical procedure: dissection with non-robotic scissors after subcutaneous infiltration and then robotic dissection (6). This surgical procedure with incision in anterior axillary line allowed good cosmetic results without scar on breast and without dorsal scar with RLDF and the endoscopic incisions had the lowest rate of NAC necrosis at 4.9% on the recent study of Daar et al. (31). We reported a strong selection of patients: even if we have patients with previous radiotherapy, patients with IBR were significantly younger (86.7% were between 41 and 74 years old), with a BMI <25 (75%), with small breast volume and a weight of specimen fewer than 300 g (58.8%) and classified as low risk for anesthesia (ASA 1–2) for 94%. In multivariate analysis, robotic surgery was not significant in terms of complications compared to non-robotic surgery (*p* = 0.263) and not significant for duration of surgery >180 min compared to a conventional surgery (*p* = 0.460). It's probably the increase experience of robotic breast-surgery in our center which allowed non-significant times of procedures as described in a recent study (6, 19).

Strengths of the present study include the relatively important number of cases compared to other series on ILBLR. We proposed a variety methods of reconstruction: LDF with traditional open technique or robotic technique (6, 19) with or without implant, retro-pectoral implant, expander tissue with a relatively low rate of complications. There is no confounding issue with the radiation treatment protocols because only one radiation therapy center was used to treat the patients. Some limitations of the present study can be underline: our cohort was not prospective without cosmetic results and quality of life analysis, we did not include a non-irradiated control group for comparison, and we don't performed pedicle or free abdominal flap. Patients who required free flap reconstruction were referred in another center.

## Conclusion

Prior BCT did not contribute to higher surgical complications in patients who received mastectomy and IBR. Salvage mastectomy with IBR is a good choice for local recurrence after BCT, particularly using flap reconstruction, without significant negative prognostic impact. NSM can be considered as a good treatment option in highly selected patients for ILBLR with or without robotic surgery for NSM and/or LDFR.

## Data Availability Statement

The raw data supporting the conclusions of this article will be made available by the authors, without undue reservation.

## Ethics Statement

The studies involving human participants were reviewed and approved by Paoli-Calmettes Institute's review board (our ethical committee). The patients/participants provided their written informed consent to participate in this study.

## Author Contributions

All authors were responsible for date collection and analysis, interpretation of the results, and writing the manuscript, seen and approved both the primary submission and any revisions and the manuscript is not in press and submitted for consideration of publication elsewhere currently, approved final version of paper for submission.

## Conflict of Interest

The authors declare that the research was conducted in the absence of any commercial or financial relationships that could be construed as a potential conflict of interest.
