# Supplementary material for: Types of Mastectomies and Immediate Reconstructions for Ipsilateral Breast Local Recurrences
Source: Front Oncol. 2020 Dec 10;10:567298. doi: 10.3389/fonc.2020.567298 (PMC7758529; doi:10.3389/fonc.2020.567298)
Supplement: Supplementary file 1 [file Table_1.docx]

**Supplementary Table 1** : Types and grades of complications according to IBR or not IBR.

|  | **Grade 1** | **Grade 2** | **Grade 3** |
| --- | --- | --- | --- |
| **Patients without IBR** |  |  |  |
| Parietal breast seroma | 28 |  |  |
| Skin necrosis | 4 | 3 | 2 |
| Hematoma | 1 | 1 | 5 |
| Infection |  |  | 2 |
| **Patients with IBR** |  |  |  |
| Dorsal seroma | 14 |  |  |
| Skin necrosis | 7 | 1 |  |
| Hematoma | 1 |  | 3* |
| Infection |  | 1 | 2 |
| implant loss |  |  | 3 |
|  |  |  |  |
| * including 1 dorsal bleeding | |  |  |
