# Supplementary material for: Types of Mastectomies and Immediate Reconstructions for Ipsilateral Breast Local Recurrences
Source: Front Oncol. 2020 Dec 10;10:567298. doi: 10.3389/fonc.2020.567298 (PMC7758529; doi:10.3389/fonc.2020.567298)
Supplement: Supplementary file 2 [file Data_Sheet_1.docx]

Supplementary data 1: Univariate analysis of factors associated with longer duration of surgery for patients with immediate breast reconstruction.

| Surgery time *(min)*  Patients with IBR | Mean | Median | CI 95% | *p* |
| --- | --- | --- | --- | --- |
| Type of Mastectomy |  |  |  |  |
| NSM | 253 | 252.5 | 212-294 | .029 |
| SSM | 189 | 148 | 148-230 |  |
| Type of Reconstruction |  |  |  |  |
| Breast implant | 112 | 93 | 95-130 | <.0001 |
| LDF RLDF | 303 | 285 | 274-332 |  |
| LDF without implant | 283 | 266 | 257-309 | .005 |
| LDF with implant | 377 | 365 | 287-466 |  |
| Period |  |  |  |  |
| P1 | 250 | 257 | 213-287 | .001 |
| P2 | 152 | 143 | 119-184 |  |
| Robotic Surgery |  |  |  |  |
| Yes | 311 | 300 | 274-349 | <.0001 |
| No | 155 | 139 | 126-183 |  |
