# Supplementary material for: Types of Mastectomies and Immediate Reconstructions for Ipsilateral Breast Local Recurrences
Source: Front Oncol. 2020 Dec 10;10:567298. doi: 10.3389/fonc.2020.567298 (PMC7758529; doi:10.3389/fonc.2020.567298)
Supplement: Supplementary file 3 [file Data_Sheet_2.docx]

Supplementary data 2: Univariate analysis of factors associated with complications for patients with immediate breast reconstruction.

| **Characteristics** | **Complications** | | ***p*** |
| --- | --- | --- | --- |
|  | No  (n = 37) | Yes  (n = 31) |  |
| Tobacco, n (%) |  |  | .030 |
| no | 33 (89.2) | 21 (67.7) |  |
| yes | 4 (10.8) | 10 (32.3) |  |
| BMI, n (%) |  |  | .061 |
| < 25 | 31 (83.8) | 20 (64.5) |  |
| ≥25 | 6 (16.2) | 11 (35.5) |  |
| Cup size, n (%) |  |  | .028 |
| A-B | 24 (64.9) | 12 (38.7) |  |
| ≥C | 13 (35.1) | 19 (61.3) |  |
| Mastectomy, n (%) |  |  | .252 |
| NSM | 15 (40.5) | 16 (51.6) |  |
| SSM | 22 (59.5) | 15 (48.4) |  |
| Reconstruction, n (%) |  |  | .011 |
| Implant | 23 (62.2) | 8 (25.8) |  |
| LDF | 11 (29.7) | 18 (58.1) |  |
| LDF + Implant | 3 (8.1) | 5 (16.1) |  |
| Surgery time, n (%) |  |  | .006 |
| <180 m | 21 (56.8) | 7 (23.3) |  |
| > 180 m | 16 (43.2) | 23 (76.7) |  |
